# Zebrafish: a convenient tool for myelopoiesis research

**DOI:** 10.1186/s13619-022-00139-2

**Published:** 2023-01-03

**Authors:** Yang-Xi Hu, Qing Jing

**Affiliations:** 1 Department of Cardiology, Changzheng Hospital, Shanghai, 200003 China; 2 CAS Key Laboratory of Tissue Microenvironment and Tumor, Shanghai Institute of Nutrition and Health, Chinese Academy of Sciences, 320 Yue-Yang Road, Shanghai, 200031 China

**Keywords:** Myelopoiesis, Hematopoietic stem/progenitor cells, Myeloid disorder, Zebrafish

## Abstract

Myelopoiesis is the process in which the mature myeloid cells, including monocytes/macrophages and granulocytes, are developed. Irregular myelopoiesis may cause and deteriorate a variety of hematopoietic malignancies such as leukemia. Myeloid cells and their precursors are difficult to capture in circulation, let alone observe them in real time. For decades, researchers had to face these difficulties, particularly in in-vivo studies. As a unique animal model, zebrafish possesses numerous advantages like body transparency and convenient genetic manipulation, which is very suitable in myelopoiesis research. Here we review current knowledge on the origin and regulation of myeloid development and how zebrafish models were applied in these studies.

## Background

Myelopoiesis is a complex process in which the myeloid progenitors, with multiple differentiation events, develop into mature myeloid cells, including monocytes/macrophages and granulocytes. The myeloid progenitor cells derive from hematopoietic stem cells (HSCs), the pluripotent adult stem cells with self-renewal and differentiation potential as well as the origin of mature blood cells in the body. The characteristics of myeloid progenitor cells (MPCs) and HSCs, such as rare number, complex differentiation pedigree, and uncertain migration in the circulation, greatly increase the difficulty of in vivo studies using conventional animal models.

Zebrafish emerged as a classical developmental and embryological model organism because of its transparent in vitro fertilization and rapid embryonic development. Thousands of zebrafish mutants with abnormal hematopoiesis were produced through forward genetic screening, showing a high degree of conservation of pathogenic genes in the process of hematopoiesis in different species (Gore et al. 2018). The high conservation of zebrafish genes provides easy access to identify genes related to the hematopoietic disorders of human beings (Robertson et al. 2016). Through different genetic means and drug screening, the expression of specific genes could be induced or inhibited in specific tissues of zebrafish, paving the way to precisely study their roles in human diseases (Kafina and Paw 2018). Fortunately, zebrafish embryos can tolerate severe anemia, myeloproliferative diseases, and other serious hematopoietic diseases that usually lead to embryonic death of mammalian models to a large extent, making it indispensable in the in vivo research of human hematopoiesis and hematological malignancies. Through reverse genetic editing, thousands of zebrafish embryos can be genetically manipulated to test the gene mutations found in human research, even in a single experiment. Through the transient overexpression of homologous genes related to human hematopoietic malignancies in zebrafish, or the construction of stable transgenic lines of these genes, the studies of acute and chronic hematopoietic diseases have been greatly facilitated.

Zebrafish has the basic myeloid cell types observed in mammals, including monocyte, neutrophil, eosinophil, mast cell, dendritic cell, and so on. The myelopoiesis process is also conserved between zebrafish and most other vertebrates. Thus, the findings from zebrafish models are possible to be directly applied to other higher vertebrates (Zang et al. 2022). At present, researchers constructed transgenic lines in zebrafish that labeled specific cell clusters to track the generation and migration of MPCs and myeloid cells during the development and under various chemical or genetic disturbances in real time. In this review, we provide an overview of the myelopoiesis research using zebrafish models and their findings.

## The myelopoiesis of zebrafish

**Fig. 1:**
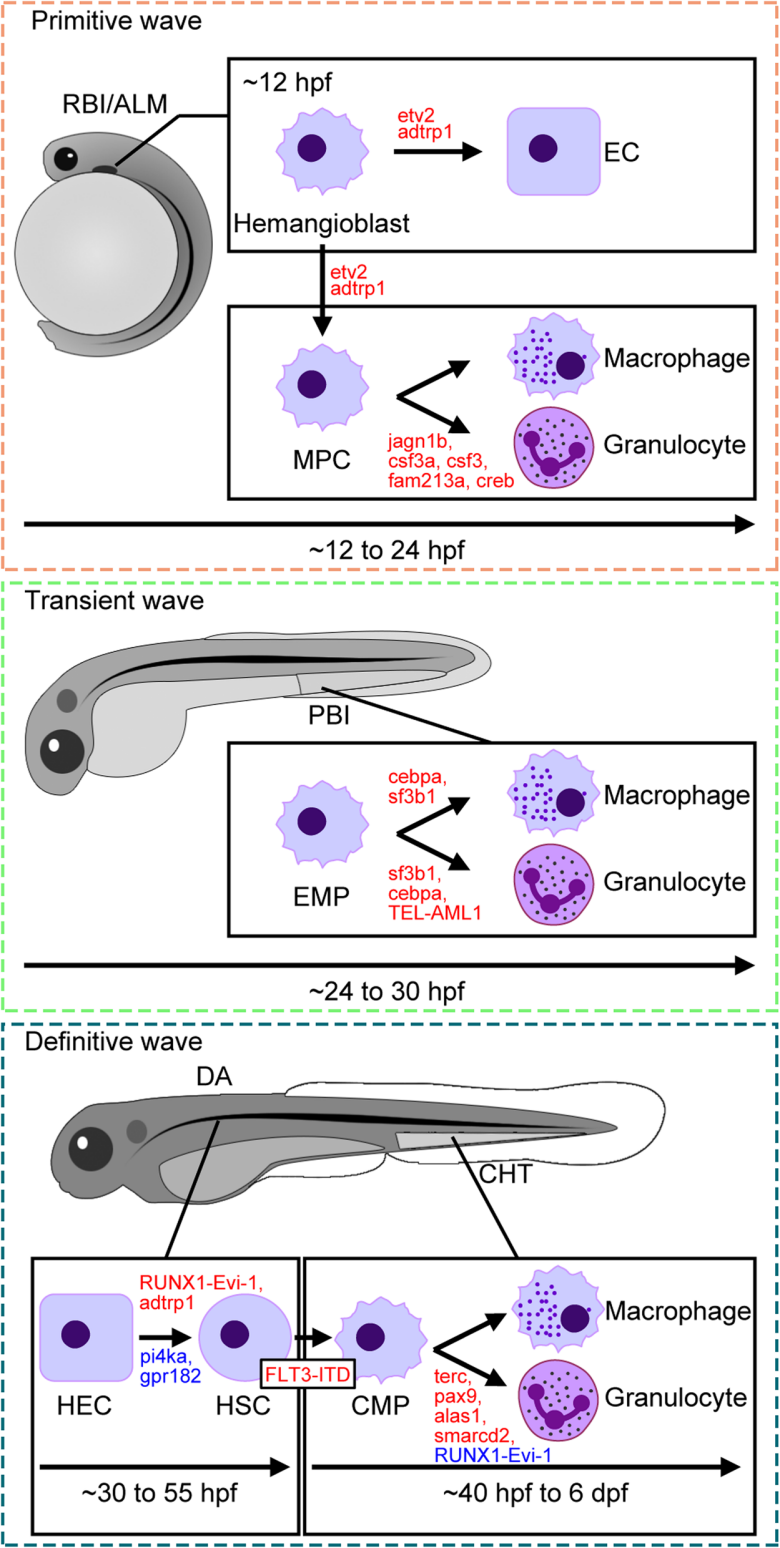
Zebrafish myelopoiesis and recently acknowledged regulators. Zebrafish myeloid cells derives from myeloid progenitor cells and hematopoietic stem cells. The sites each hematopoietic wave takes place are indicated together with timelines, as are some of the recently reported regulators of zebrafish myelopoiesis. The promotive and suppressive regulators are labelled in red and blue, respectively. RBI, rostral blood island; ALM, anterior lateral mesoderm; EC, endothelial cell; MPC, myeloid progenitor cell; PBI, post blood island; EMP, erythroid-myeloid progenitor; DA, dorsal aorta; CHT, caudal hematopoietic tissue; HEC, hemogenic endothelial cell; HSC, hematopoietic stem cell; CMP, common myeloid progenitor

Figure 1 depicts the process of zebrafish myelopoiesis and some of the recently acknowledged regulators. The primitive hematopoiesis of zebrafish begins at 11 hours post fertilization (hpf) and mainly produces primitive macrophages, neutrophils, and erythrocytes (Detrich 3rd et al. 1995; Le Guyader et al. 2008). But primitive myeloid cells and erythrocytes have different developmental origins. The anterior lateral mesoderm (ALM) locates a kind of precursor cells with the dual potential to generate hematopoietic cells, and vascular endothelial cells (ECs) called hemangioblasts (Vogeli et al. 2006). Single-cell fate tracing of late blastocysts and gastrula of zebrafish proved that individual hemangioblasts could produce hematopoietic cells and ECs at the same time (Vogeli et al. 2006). Etv2/etsrp, an ETS transcription factor, is a key regulator of hemangioblasts as well as one of the earliest markers for the progenitors of ECs and hematopoietic cells (Craig et al. 2015; Sumanas and Lin 2006). At around 12 hpf, the myeloid precursors, the origin of primitive macrophages, are differentiated from hemangioblasts. Subsequently, myeloid precursors begin to migrate and differentiate into macrophages and granulocytes in the rostral blood island (RBI) (Bennett et al. 2001). Primitive hematopoietic cells produce macrophages and neutrophils that initiate innate immunity. Primitive neutrophils have little phagocytic capacity yet response to infection or environmental stress (Le Guyader et al. 2008). The differentiation of hemangioblast into myeloid precursor is regulated by multiple factors and signaling pathways. For instance, the *FGF/MAPK* signal pathway (Faloon et al. 2000), the *VEGF/Flk1* signal pathway (Cao and Yao 2011), and the *BMP* signal pathway (Kennedy et al. 2007) are important for the development and differentiation of hemangioblasts. Dysfunction of the key components in these pathways will lead to the failure of HPSC generation and affect the expression of hematopoietic marker genes such as *scl*, *gfi1*, and *lmo2*.

The definitive hematopoiesis could be subdivided into two waves. At about 24 hpf, there is a transient hematopoietic wave in the posterior blood island (PBI) to produce erythroid-myeloid progenitors (EMPs). These cells do not have the ability of self-renewal and can only differentiate into myeloid and erythroid blood cells (Bertrand et al. 2007). At 28-30 hpf, the second wave of the definitive hematopoietic process begins to occur (Bertrand et al. 2007). Different from the transient wave, the definitive wave sustains into the adult stage of zebrafish, producing HSCs, which differentiate into lymphocytes, myeloid cells, and erythroid cells. In the ventral wall of the dorsal aorta (VDA), which is homologous to the aorta-gonad-mesonephros (AGM) region of mammals, hemogenic endothelial cells (HECs) differentiate into HSCs through the process of endothelial hematopoietic transition (EHT) (Kissa and Herbomel 2010). The transcription factor c-myb is one of the most important regulators of myelopoiesis, whose inhibition will result in impaired egression of HSPC from the VDA while hyperactivity leads to abnormal granulocyte expansion (Liu et al. 2017; Zhang et al. 2011). Nascent HSCs robustly regenerate after selective injury, yet do not contribute to early lymphopoiesis and myelopoiesis. Instead, HSC-independent blood progenitors give rise to embryonic lymphomyelopoiesis (Ulloa et al. 2021). From 48 hpf, *c-myb* positive cells can be detected in the caudal hematopoietic tissue (CHT), which is functionally homologous to mammalian fetal liver and contributes to the growth and differentiation of HSCs (Murayama et al. 2006). Through real-time imaging, it was observed that HSCs migrated to the CHT through circulation, after which they sprouted out and stayed in the microenvironment around ECs (Tamplin et al. 2015). After close contact with ECs, HSCs divide in an asymmetric manner, which seems to depend on the cellular niche of mesenchymal stem cells (MSCs) (Morrison and Kimble 2006; Morrison and Spradling 2008). When the embryo develops to 4 dpf, HSCs gradually migrate to the kidneys and begin to settle down. The kidney is equivalent to the bone marrow of mammals and becomes a definitive hematopoietic organ, maintaining the whole blood system and producing blood cells required for the whole life stage, including myeloid cells (Thompson et al. 1998). In the process of myelopoiesis, a part of macrophages migrates to specific tissue and then specializes into resident macrophages. The origin, function, and fate of various resident macrophages are yet elusive. Microglia, the CNS resident macrophages, is once supposed to originate from a single source, the yolk sac. Using light-induced high temporal-spatial resolution fate mapping, Xu et al. revealed that in zebrafish, the embryonic/larval microglia arise from the RBI, whereas the adult microglia arise from the VDA, regulated by *runx1* but not *c-myb*, which is different from the embryonic/larval microglia (Xu et al. 2015).

Besides developmental myelopoiesis, there exists emergency myelopoiesis, which generates new neutrophils and other myeloid cells following infection or inflammation (Basu et al. 2000; Ueda et al. 2009). The emergency myelopoiesis is associated with the expansion of HSPCs and MPCs. To some extent, HSPC differentiation could be enhanced by infective or inflammatory cues. However, excessive burden causes the apoptosis of HSPCs and therefore suppresses emergency myelopoiesis (Abidin et al. 2017). As to be described in detail below, the transcription factor pu.1 plays a main promotive role in physiological macrophage development (Hou et al. 2016). However, in emergency monopoiesis, pu.1 is suppressive, which may partly explain the different patterns of emergency myelopoiesis in different scenarios. In emergency myelopoiesis, some proinflammatory cytokines regulate various hematopoietic compartments. For instance, IFN-γ is known to negatively regulate HSC proliferation until recent studies discovered that IFN-γ also promotes HSC formation and consequent myelopoiesis (Qin and Zhang 2017). Macrophage-derived il1β actives nf-κb and c/ebpβ, two fast-reactive transcription factors, rapidly. In an LPS-induced systemic infection zebrafish model, the macrophage is the first cell type to detect LPS and secret il1β, which is further responded to by MPCs, rather than HSPCs, expanding myelopoiesis through the synergistic cooperation of nf-κb and c/ebpβ (Wei et al. 2020).

## Using zebrafish to explore the regulation of myelopoiesis

Zebrafish myelopoiesis is complicated yet precisely regulated by both genetic and extrinsic factors, as described in a precious review before (Xu et al. 2012). Firstly, the specification and differentiation of myeloid precursors and MPCs are controlled by various transcription factors, namely *runx1*, *pu.1*, *irf8*, *gata* family, *lmo2*, *scl*, and others in a spatially and temporally coordinated manner. Table 1 summarizes some of the important transcription factors in myelopoiesis and myeloid disorders. In addition, the regulatory factors upstream these transcription factors, such as *tif1g*, *bik1f*, and *gfi1.1*, are also found effective in myeloid-erythroid lineage choice of primitive hematopoietic progenitors. Secondly, complex signaling pathways regulate the differentiation of hemangioblast into hematopoietic cells, including the *cloche/etsrp/scl* pathway and the *Bmp/Alk8* pathway. Thirdly, extrinsic factors like granulocyte colony-stimulating factor (GCSF) affect the development and migration of myeloid cells (Meier et al. 2022). Given these known, in this section, we selected and reviewed several recent progresses in the understanding of myelopoiesis gained from zebrafish models.

**Table 1:** Important transcription factors in myelopoiesis and myeloid disorders

Transcription factor	Family	Genetic manipulation	Hematopoietic phenotype	Reference
pu.1	ETS	Knockout	Immature granulocytes	(Sun et al. 2013)
spi-b	ETS	Knockdown	Decreased neutrophils and macrophages	(Bukrinsky et al. 2009)
runx1	Runt	Knockout	Immature neutrophils	(Huang et al. 2021)
C/EBPα	Runt	Knockout	Decreased macrophages and neutrophils	(Dai et al. 2016; Hockings et al. 2018)
C/EBPβ	Runt	Knockdown	Suppressed emergency granulopoiesis	(Hall et al. 2012)
C/EBPδ	Runt	Knockdown	Increased myeloid differentiation	(Tregnago et al. 2016)
irf8	IRF	Knockout	Impaired macrophage specification and longevity	(Tamura et al. 2005)
gata1	GATA	Knockout	Reduced eosinophils, morphologically abnormal mast cells, impaired dendritic-cell differentiation	(Gutiérrez et al. 2007; Migliaccio et al. 2003; Yu et al. 2002)
gata2a	GATA	Knockout	Reduced primitive monocyte/macrophages	(Peña et al. 2021)
gata2b	GATA	Knockout	Reduced definitive HSPCs and neutrophils	(Gioacchino et al. 2021; Peña et al. 2021)
gata4/5/6	GATA	Knockdown	Reduced definitive granulocytes and macrophages	(Jia et al. 2019)
SOX4	HMG	Overexpression	Enhance MPC proliferation	(Sandoval et al. 2012)
pax9	Pax	Knockout	Reduced neutrophils	(Pak et al. 2021)
scl	HLH	Knockdown	Impaired monocytes and macrophages	(Dooley et al. 2005)
lmo2	LIM	Knockdown	Reduced granulocytes and macrophages	(Patterson et al. 2007)
c-myb	Myb	Knockout	Immature neutrophils	(Jin et al. 2016)

### Transcriptions factors and their regulation

The well-known hematopoietic transcription factor runx1 regulates neutrophil maturation in zebrafish primitive myelopoiesis. Huang et al. reported that runx1, synergistically with another hematopoietic transcription factor, c-myb, controls myelopoiesis through activating downstream genes involving neutrophil maturation (Huang et al. 2021). In definitive hematopoiesis, runx1 shows a dynamic expression pattern during the specialization of HSCs. In zebrafish, *runx1* deficiency will cause the termination of EHT, and immature HSCs will be rapidly broken into fragments (Kissa and Herbomel 2010). When the cofactor cbfβ of runx1 is knocked out, HSCs cannot move out from the dorsal aorta (DA), and definitive hematopoiesis cannot occur (Bresciani et al. 2014). In three new zebrafish lines with *runx1* deficiency, Bresciani et al. observed that runx1 and gata2 act redundant roles for HSC production acting as each other’s safeguard (Bresciani et al. 2021).

The CCAAT/enhancer-binding proteins (C/EBPs) are a group of transcription factors that play important roles in developmental myelopoiesis conserved between zebrafish and other mammals. C/EBPα maintains the steady-state granulopoiesis while C/EBPβ is required for stress-induced granulopoiesis. Hockings et al. reported that biallelic mutations in both C and N terminals of c/ebpα caused severely impaired myelocyte and monocyte maturation (Hockings et al. 2018; Hockings et al. 2016). Dai et al. reported that c/ebpα-deficient zebrafish mutants manifested a lack of macrophages and neutrophils, which is caused by the cell cycle arrest of MPCs. They found that the function of c/ebpα on myelopoiesis probably requires the corporation with runx1 (Dai et al. 2016). C/EBPβ is indispensable for emergency granulopoiesis (Hall et al. 2012) and responsible for expanded myelopoiesis in many hematological malignancies like chronic myeloid leukemia (CML) (Hirai et al. 2015). C/EBPδ expression increased in a zebrafish model overexpressing CREB, a proto-oncogene frequently overexpressed in AML patients, in the myeloid lineage, causing differentiation arrest of myelopoiesis (Tregnago et al. 2016). C-myb is one of the earliest transcription factors expressed in HSCs, whose loss-of-function leads to severe defects in neutrophil differentiation in zebrafish. C-myb synergistically functions with cebp1, the zebrafish functional homolog of mammalian C/EBPε, directly targets *lyz* (encodes a key protein component in neutrophils), thereby promoting the maturation of neutrophils (Jin et al. 2016). PTEN, a famous tumor suppressor, regulates definitive myelopoiesis in zebrafish via promoting *c/ebpα* and suppressing the *PI3K/mTOR* pathway. *Pten*-deficient zebrafish larvae manifest reduced apoptosis and strongly expansive definitive myelopoiesis while the maturation and immune response of myeloid cells are blocked (Dong et al. 2014).

Pu.1 is an essential ETS-family transcription factor for generating myeloid precursors and their fate determination during myelopoiesis. The suppression of *pu.1* results in increased immature myeloid cells (Sun et al. 2013), while its upregulation induced by various factors such as histone demethylase Jmjd3 leads to enhanced myeloid commitment and repressed erythroid commitment during both the primitive and definitive myelopoiesis (Yu et al. 2018). Even though it is well known that *pu.1* is indispensable in myelopoiesis, whether it is differentially required for the primitive and definitive stages remains unknown. In zebrafish, Yu et al. noticed that the VDA-derived macrophage development depends on the synergistic function of *pu.1* and *spi-b* (another ETS transcription factor). While in the RBI-derived macrophage development, *pu.1* acts upstream *spi-b*. In both waves of macrophage development, irf8 serves as the common downstream factor (Yu et al. 2017). The determination of myeloid fates in HSCs depends mainly on the cross-inhibitory relationship between *gata1* and *pu.1*. Inflammasome mediates the cleavage of *gata1*, not only suppresses erythropoiesis (De Maria et al. 1999), but also shifts the balance of *gata1* and *pu.1*, in turn, regulates myelopoiesis in zebrafish (Tyrkalska et al. 2019). The antagonist-induced inhibition of inflammasomes impaired both physiological and demand-driven myelopoiesis and rescued the phenotypes in zebrafish neutrophilic dermatosis and Diamond-Blackfan anemia models (Tyrkalska et al. 2019).

Interferon regulatory factor 8 (irf8) is an evolutionarily conserved transcription factor differently required in myelopoiesis at different developmental stages (Holtschke et al. 1996; Tamura et al. 2005). Irf8 is essential for macrophage development during primitive and transient definitive hematopoiesis, but not necessary in definitive myelopoiesis starting at 5-6 dpf. Irf8-deficient zebrafish larvae have more neutrophils and excessive cell death in *pu.1*-expressing myeloid cells, indicating that irf8 is critical to macrophage specification and longevity. Interestingly, besides the reduction in number and immaturities, tissue-resident macrophages become apparent in irf8 mutants along with the development, except microglia (Shiau et al. 2015). The interferon regulatory factor 2 binding protein 2a (irf2bp2a), a ring finger protein, regulates neutrophil differentiation in zebrafish, functioning as a ubiquitin E3 ligase. Gao et al. reported that irf2bp2a targets *gfi1aa* for proteasomal degradation, while its mRNA expression is, in turn, passively regulated by gfi1aa. These form a negative feedback loop in zebrafish myelopoiesis (Gao et al. 2021). The interferon regulatory factor 2 binding protein 2b (irf2bp2b) is a negative transcription regulator. During the definitive myelopoiesis in zebrafish, irf2bp2b influences the fate determination of MPCs in favor of macrophages, under the control of C/EBPα (Wang et al. 2020).

GATA transcription factors comprise a family of zinc finger proteins that bind the consensus DNA sequence (T/A)GATA(A/G). Among them, GATA1 is considered as a key regulator of erythropoiesis. Interestingly, GATA1 is also a regulator of myelopoiesis. For example, the GATA1 loss-of-function was found to cause the reduction of eosinophils. GATA1 deficiency was also reported to induce the morphological abnormality of mast cells and impaired differentiation of dendritic cells. In addition, an acquired N-terminal truncating mutation in GATA1 during fetal life has been reported to cause a preleukaemic condition known as transient abnormal myelopoiesis (TAM) (Garnett et al. 2020). In fact, GATA1 and PU.1 regulate erythropoiesis versus myelopoiesis by antagonizing each other (Campbell et al. 2021). Zebrafish express two *gata2* homologous genes, *gata2a* and *gata2b*. *Gata2a* began to express in hemangioblasts at 11 hpf, while *gata2b* was specifically expressed in HECs and was crucial to the production of HSCs (Butko et al. 2015). *Gata2a* is vital for primitive myelopoiesis, while *Gata2b* mainly supports definitive myelopoiesis, particularly the development of neutrophils (Peña et al. 2021). *Gata2b* mutants have impaired myeloid lineage differentiation raised in HSPCs. *Gata2b*-deficient HSPCs showed impaired myeloid transcriptional program and increased expression of lymphoid genes (Gioacchino et al. 2021). In addition, gata4/5/6 are all reported to promote primitive myelopoiesis via downregulating miR-210-5p expression (Jia et al. 2019).

SOX4 is known as a transcription factor that belongs to the high-mobility group (HMG) domain superfamily. SOX4 is required in lymphopoiesis (Mallampati et al. 2014) and is reported to be expressed and contributive to the development of multipotent progenitors (Zhang et al. 2014). Yet, how SOX4 affects myelopoiesis remains elusive. Boyd et al. reveal that SOX4 in the murine myeloid cell line 32Dcl3 blocks cytokine-induced granulocyte maturation (Boyd et al. 2006). Through direct binding to the promoter of *CREB* and inducing *CREB* expression, SOX4 up-regulates the target genes of CREB and promotes the proliferation and self-renewal of MPCs (Sandoval et al. 2012). Lu et al. established a transgenic zebrafish model overexpressing human *SOX4* in the myeloid lineage. The mutants developed expanded myelopoiesis with dedifferentiation in kidney marrow at 5 months of age and severely distorted kidney structure with excessive MPCs (Lu et al. 2017).

Paired Box (Pax) transcription regulator family members are involved in many diseases related to organ development processes. Recently, one of its members, Pax9, was found to be indispensable for granulopoiesis without affecting erythropoiesis in zebrafish. *Pax9*-deficiency led to decreased neutrophils and the expression of neutrophil-specific markers in both developmental and emergency granulopoiesis (Pak et al. 2021).

### Chromatin regulators

The SMARCD2, also known as BAF60b, is a component of the SWI/SNF complex in HSCs and other hematopoietic cells and regulates the differentiation of myeloid-erythroid progenitor cells (Schim van der Loeff et al. 2021; Witzel et al. 2017). In vivo experiments using zebrafish and mice proved that SMARCD2 is required in neutrophil differentiation, with high evolutionary conservation. SMARCD2 recruits C/EBPε, which further targets the promoters of many granule genes such as *CAMP* and *SERPINA1*, lactoferrin (*LTF*), and matrix metalloproteinase 8/neutrophil collagenase (*MMP8*). Notably, SMARCD2 takes stage-specific roles in granulopoiesis. During granulosis, neutrophils pass through various developmental stages including CD45^+^Lin^−^Sca-1^+^c-Kit^+^ LSK cells, CD45^+^Lin^−^Sca-1^−^c-Kit^+^CD34^+^CD16/32 (FCGR)^int^ common myeloid progenitors (CMPs), and CD45^+^Lin^−^Sca-1^−^c-Kit^+^CD34^+^CD16/32 (FCGR)^high^ granulocyte–macrophage progenitors (GMPs) or CD45^+^Lin^−^Sca-1^−^c-Kit^+^CD34^−^CD16/32 (FCGR)^low^ megakaryocyte–erythroid progenitors (MEPs). As reported by Witzel et al., SMARCD2 acts as a transcriptional suppressor in immature neutrophils (LSK and CMP cells), while it plays a transcriptional activator in further differentiated stages (MEP and GMP cells) (Witzel et al. 2017).

Ring finger protein 4 (RNF4) also takes part in the regulation of granulopoiesis. The neutrophils were largely decreased in rnf4-deficient zebrafish larvae during both primitive and definitive myelopoiesis. Mechanistically, the hypermethylation of c/ebpα promoter by SUMOylated DNA methyltransferase 1 (DNMT1) led to granulopoiesis defect in rnf4-deficient zebrafish (Wang et al. 2018a). Besides DNMT1, another methyltransferase, smyd5, suppresses the excessive expressions of primitive and definitive myelopoietic genes, such as *pu.1*, *mpx*, *l-plastin*, and *c-myb* (Fujii et al. 2016).

### Extrinsic factors and their regulation

The GCSF drives the proliferation and differentiation of granulocytes, monocytes, and macrophages from HSPCs. The two GCSF ligands in zebrafish, gcsfa and gcsfb, are less similar in sequences while closely resemble each other in predicted ligand/receptor interaction sites and structures. Both bind to the GCSF receptor and promote primitive and definitive development of myeloid cells, and both support the specification and proliferation of HSCs (Stachura et al. 2013). Colony-stimulating factor 1 receptor (Csf1r) also regulates microglia development in vertebrates. Csf1r, or Fms, together with pu.1, function synergistically to support the development of osteoclasts and myeloid cells (Liu et al. 2020). Among them, pu.1 masters osteoclastogenesis, whereas Fms promotes osteoclast maturation. The two zebrafish *csf1r* paralogous genes, *csf1ra* and *csf1rb*, play different roles in myelopoiesis. Ferrero et al. reported that csf1ra, but not csf1rb, is essential for primitive myelopoiesis in zebrafish (Ferrero et al. 2021). For the HSC-derived myelopoiesis in definitive and adult hematopoiesis, it is promoted by csf1rb with reduced macrophages, including microglia (Ferrero et al. 2021). Single-cell RNA sequencing analysis for adult whole kidney marrow (WKM) hematopoietic cells also revealed that *csf1rb* is expressed mainly by MPCs, in a nonoverlapping pattern with *csf1ra* (Hason et al. 2022).

Bone morphogenetic proteins (BMPs) are a group of growth factors regulating HSC differentiation and myelopoiesis. The receptor type Smad proteins, namely Smad1, Smad5, and Smad9, help to transduce nuclear BMP signals. Among them, transcription initiations of smad1 and smad9 are mutually repressive yet indispensable in myelopoiesis, while both are direct downstream of Smad5 (Wei et al. 2014).

The suppressors of cytokine signaling (SOCS) family consists of eight proteins that antagonize the signaling of STAT proteins, whose members are reported by multiple studies to regulate the process of developmental myelopoiesis and progression of myeloid leukemia. The zebrafish model overexpressing SOCS1 in MPCs demonstrated effaced and distorted kidney or spleen structure with increased MPCs and myelopoiesis in kidney marrow (Hou et al. 2017). Among all SOCS family members, socs3b is the most expressed in neutrophils. During 35-48 hpf, the time window for granulation, *socs3b* was usually downregulated, indicating socs3b’s regulatory role in myelopoiesis (Banks et al. 2021). The expression of *socs3b* could be upregulated in neutrophils with compound *tet2/3* mutant, indicating that zebrafish tet enzymes demethylate and destabilize *socs3b* mRNA during granulation, thereby maintaining the cytokine signaling to support physiological neutrophil maturation (Banks et al. 2021).

Stag1 and Stag2, two components of cohesin multiprotein complex, corporately regulate the production of hemangioblasts. The depletion of both *stag1a* and *stag2b* in zebrafish results in erythropenia in primitive hematopoiesis. Homozygous loss of *stag1a* leads to expanded LPM with increased *scl*-positive cells and increased *pu.1*-positive cells, indicating the skewing toward primitive myelopoiesis (Ketharnathan et al. 2020).

The mutations of isocitrate dehydrogenase 1/2 (IDH1/2) have been identified in ~ 30% of cytogenetically normal AML patients. These two enzymes are involved in the citric acid cycle in intermediary metabolism. Zebrafish model carrying human IDH1-R132H mutation, which is frequently identified in AML patients, also developed AML-like phenotypes (Shi et al. 2015). Shi et al. found that zebrafish *idh1* deficiency induced blocked myeloid differentiation, characterized by the increased expression of *pu.1* and decreased expressions of *mpo*, *l-plastin,* and *mpeg1.* Furthermore, *idh1* deficiency led to the significant reduction of *runx1* and *c-myb* expression in the VDA and the CHT region at definitive hematopoiesis. Meanwhile, the supplement of *idh2* mRNA failed to rescue the impaired myeloid differentiation induced by *idh1* deficiency, which means there exists no redundancy between the effects of idh1/2 on myelopoiesis (Shi et al. 2015).

G protein-coupled receptor and their ligands play multifunctional roles in myelopoiesis and erythropoiesis. For example, the deficiency of a G-protein-coupled ADP receptor p2y12 was reported to result in excessive primitive erythropoiesis in zebrafish embryos, mainly attributed to enhanced expression of *gata1* (Li et al. 2021). Another two G-protein-coupled receptors, Gpr56 and Gpr97, were redundantly functioning in normal HSC development and differentiation (Maglitto et al. 2021). Expressed in HECs, the orphan G-protein coupled receptor 182 (Gpr182) suppressed the excessive definitive myelopoiesis by regulating the LTB4 biosynthesis pathway (Kwon et al. 2020).

The 5-aminolevulinate synthase 1 (ALAS1) is a rate-limiting enzyme of the biosynthesis of heme (iron protoporphyrin IX, a prosthetic group on hemoproteins like hemoglobin and myoglobin). Another isoform, ALAS2, mainly expressed in erythroid cells, regulating the production of heme for hemoglobin synthesis. ALAS1 ubiquitously expressed in various cell lineages including neutrophils, implying that heme not only is indispensable in erythropoiesis but also may affect myelopoiesis. The deficiency of *alas1* in zebrafish, which leads to reduced production of heme, impairs the maturation of neutrophils (Lian et al. 2018). This finding also indicates the existence of an intricate crosstalk exists between erythropoiesis and myelopoiesis.

Tissue factor (TF) and the TF pathway have long been known to regulate hematopoiesis. *Adtrp* encodes a regulating protein for the inhibitor of the androgen-dependent TF pathway (TFPI). Recently, a novel *adtrp1*-*tfpi* axis has been identified to regulate the processes of both primitive and definitive myelopoiesis. Among the two paralogs of *adtrp* in zebrafish (*adtrp1* and *adtrp2*), *adtrp1* promotes the expression of primitive myelopoietic markers like *pu.1*, *mpo*, and *l-plastin*, and supports HSC specification in definitive myelopoiesis (Wang et al. 2018b).

### Noncoding RNAs

Noncoding RNAs are emerging regulatory factors for myelopoiesis, including lncRNAs (Qiu et al. 2021) and circRNAs (Dostalova Merkerova et al. 2020). In addition, many microRNAs have been found to be regulators or effectors of myelopoietic transcription factors (Kim et al. 2019). An example in zebrafish is that the depletion of miR-462/miR-731 decreased erythroid cell number while increased myeloid cell expansion at 48 hpf, indicating their regulatory role in myeloid-erythroid differentiation. Mechanistically, miR-462/miR-731 regulates the *pu.1*-dependent primitive myelopoiesis through targeting *etsrp/scl* pathway. miR-462 and miR-731 also affect *BMP/Smad* signaling in driving primitive myelopoiesis in the ALM (Huang et al. 2019). miR-142-3p is indispensable for the definitive granulosis, whose deficiency leads to hypermaturation of neutrophils. The hypermature neutrophils demonstrated a larger size and declined nucleocytoplasmic ratio. The activation of the *IFN-γ* signaling pathway, for instance, the upregulation of *stat1a* and *irf1b*, is responsible for the impaired myelopoiesis caused by miR-142-3p deficiency (Fan et al. 2014). Downstream of transcription factors gata4/5/6, miR-210-5p inhibits primitive myelopoiesis through silencing *foxj1b* and *slc3a2a* (Jia et al. 2019). High expression of miR-129 in myeloid cells tends to favor granulocyte maturation, while lower expression favors monocyte maturation. This is because miR-129 directly suppresses the expression of runx1, which promotes monocyte differentiation yet represses granulocyte differentiation. Interestingly, runx1 in turn affects miR-129 expression at the transcriptional level, forming a feedback regulatory loop (Zhao et al. 2017). miR-223 is up-regulated between 4 and 6 dpf and from 30 dpf to adulthood in the head kidney, a well-described hematopoietic organ in zebrafish considered the equivalent to the bone marrow of mammals, and regulates the seeding of HSCs in the head kidney and definitive hematopoiesis in zebrafish. Due to the evolutional conservative functions between zebrafish and mammalian miR-223, it is speculated that *MEF2C* and *IGF1R* genes are most likely miR-223 targets in zebrafish hematopoiesis, although lacking further validation (Roberto et al. 2015). miR-191 possibly contributes to myelomonocytic differentiation in zebrafish either, because after hsa-miR-191 was microinjected into the fertilized eggs, the spatiotemporal expression of L-plastin at 24 hpf was specifically up-regulated (Sun et al. 2017).

Telomerase RNA (terc) is able to bind specific DNA sequences, recruits RNA polymerase II, and regulates the expression of myeloid genes (García-Castillo et al. 2021). This function of terc is discovered using a zebrafish *terc* mutant with the CR4-CR5 domain mutation found in patients with dyskeratosis congenita (DC) in vivo and independent on the telomerase catalytic subunit. Telomerase RNA also has a non-canonical function in regulating zebrafish myelopoiesis. Depletion of zebrafish telomerase RNA component (TR) leads to myelopoietic defects without affecting HSC development, in a form independent of telomere length and telomerase activity. This effect also depends on the GCSF and macrophage-stimulating factor (MCSF) mediated regulation of *pu.1* and *gata1* expression (Alcaraz-Pérez et al. 2014).

## Recent zebrafish mutant models of myelopoietic disorders

Various zebrafish mutants mimicking human disorders of myelopoiesis have been established (Baeten and de Jong 2018; Xu et al. 2012). In this section, we summarized some of the most recently applied ones. FAM213A has been identified to be associated with a worse prognosis of AML. A zebrafish *fam213a* mutant was used to in vivo validate its role in developmental myelopoiesis, which is a suppressor of p53 (Oh et al. 2020). The Tet methylcytosine dioxygenase 2 (TET2) is generally believed to be involved in the occurrence of hemopathy like myelodysplastic syndrome (MDS) or AML. Recently, Rajan et al. reported that zebrafish larvae with somatic *tet2* loss-of-function mutation demonstrated reduced MPCs, neutrophils, monocytes, and mast cells in definitive myelopoiesis. The emergency myelopoiesis of *tet2* mutants was also impaired, characterized by the leukemia-like excessive production of naïve myeloid cells (Rajan et al. 2022). The *BCR-ABL1* fusion gene is a characteristic of Philadelphia Chromosome (Ph) + CML. Zizioli et al. recently established a new fish line BCR-ABL1pUAS:CFP/hsp70-Gal4, expressing the human *BCR-ABL1*. The larvae carrying the human *BCR-ABL1* fusion gene presented enhanced embryonic myelopoiesis, increased myeloid cells, and suppressed apoptosis. Further study revealed that *BCR-ABL1* induced upregulated myeloid makers like *lmo2*, *pu.1*, and *mpx*, and downregulated erythropoietic markers, affecting hematopoietic cells proliferation in the CHT (Zizioli et al. 2021). Irf8 not only affects the fate determination of myeloid cells in developmental myelopoiesis but also of those in the progression of myeloid neoplasia. Myeloproliferative neoplasm (MPN) is characterized by increased myeloid precursors. *Irf8* mutant zebrafish developed an MPN-like disease with excessive myelopoiesis. One mechanism, irf8 suppressed the *Mertk* pathway in the hematopoietic cells. Thus, irf8 also acts as an evolutionarily conserved neoplastic suppressor (Zhao et al. 2018). A Noonan syndrome (NS)-like phenotype could be modeled in *Shp2*^*D61G*^ mutant zebrafish. *Shp2*^*D61G*^ mutant larvae demonstrated defective heart function and juvenile myelomonocytic leukemia (JMML)-like MPN, manifesting excessive myelopoiesis. The *Shp2*^*D61G*^ MPCs showed an increased inflammatory response, which may be responsible for this myelopoietic disorder (Solman et al. 2022). The gene *son* encodes an RNA splicing factor and is associated with Down syndrome related hematopoietic disorders. The *son*-deficient zebrafish manifested normal colony-forming capability of HSPCs, while embryonic myelopoiesis and erythropoiesis were both impaired (Belmonte et al. 2021). Ribosomal dysfunction underlies a category of diseases called ribosomopathies in humans, which is characterized by impaired hematopoiesis. Consistently, ribosome biogenesis gene deficiency causes hematopoietic defects in zebrafish (Oyarbide et al. 2019). Several studies revealed that impaired ribosome biogenesis produces excess free ribosomal proteins, which protects p53 from degradation by the E3 ubiquitin ligase MDM2 (Dai and Lu 2004; Dai et al. 2004). There are also p53-independent cell apoptosis and cell proliferation arrest described in ribosomal dysfunction-induced hematopoietic defects. For example, the deficiency of the *LTV1 ribosome biogenesis factor* (*ltv1*^*Δ14/Δ14*^), a non-ribosomal factor required for the processing of the 40S ribosomal subunit, leads to defective ribosomal biogenesis and impaired definitive hematopoiesis without affecting primitive hematopoiesis. This defect is attributed to decreased proliferation of HSPCs and independent of p53 (Zhang et al. 2021).

Through optimized inter-specific spermatogonial stem cells transplantation (SSCT), Zhang et al. recently successed in the acquirement of sperms carrying edited genome derived from a *gobiocypris rarus* honor from zebrafish recipients (Zhang et al. 2022). It is exciting that if this technology is further improved, it may provide a possibility to construct the zebrafish mutants with specific gene mutations leading to hematopoietic diseases found in any other species. This will enlarge the mutant library of zebrafish with myelopoietic and general hematopoietic disorders on an unprecedented scale.

## Limitations and prospects

Even though zebrafish as model organisms for myelopoiesis have been studied for decades, the extremely small size and complicated structure of the zebrafish model still bring great challenges to the in-depth research at the gene level and the cell level. First, the traditional transcriptome sequencing technology is based on multicellular tissue samples, which describes the mean value of signals in a pile of cells and loses the information of cell heterogeneity. It is extremely difficult to separate the tissues and organs of zebrafish embryos and larvae, so the transcriptome sequencing was usually performed on whole-body homogenate of zebrafish embryos and larvae, which further expanded the disadvantage of traditional transcriptome sequencing. The single-cell sequencing technology, which can detect the heterogeneous information of hybrid samples, solves this problem well. In 2021, a full-coverage, single-cell–resolution fate map of zebrafish early foregut endoderm was established through the labeling of endodermal progenitor cells and tracing of its descendant cells (Yang et al. 2021). Through single-cell sequencing, the dynamic state of cell composition and hematopoietic gene expression, as well as the heterogeneity and interaction with niche components of HSPC in the CHT of zebrafish was also depicted (Xia et al. 2021). These studies greatly improve myelopoiesis studies using zebrafish models in both depth and breadth. Second, the progress of microscopic imaging techniques may further facilitate myelopoiesis research using zebrafish models. In 2017, using in vivo long-term two-photon imaging, researchers directly observed the lung microcirculation in a megakaryocyte-specific fluorescent mouse line, identifying the lungs as a huge reservoir for HSPCs (Lefrancais et al. 2017), which has been ignored for nearly a century. If similar imaging technology had been used in similar studies using zebrafish models, this phenomenon might have been discovered much earlier because it is very easy to find the ectopic origin of hematopoietic cells in the long-term observation of transparent zebrafish embryos. Fortunately, a few researchers have noticed this. For example, an ectoderm-derived immune cell population, metaphocytes, has been identified in the epidermis of zebrafish (Lin et al. 2019). By using epidermis- or mesoderm-driven CreERT2 transgenes and long-term confocal imaging, Lin et al. provided convincible evidence for the existence of metaphocytes, which are highly similar to conventional Langerhans cells in transcriptome, morphology, and anatomic location, yet have different origins and functions (Lin et al. 2019). Unlike conventional myeloid cells, endoderm-derived metaphocytes cannot phagocytize apoptotic cells and invading bacteria, but are able to directly obtain soluble antigens in the environment by forming a transepithelial protrusion that passes through keratinocytes on the surface of the skin and transmit the antigens to conventional resident macrophages through apoptosis/phagocytosis axis. Although metaphocytes were initially observed in the epidermis and considered to be derived from ectoderm, in a subsequent study, they were also observed in gills and intestine, and proved to be derived from endoderm (Lin et al. 2020). These studies reveal a general existence of non-hematopoiesis-derived immune cells in zebrafish and challenge the prevalent view that resident immune cells originate exclusively from the common hematopoiesis process.

There are other defects in zebrafish models limiting their application in myelopoiesis studies. For example, flow cytometry is a widely used technique to isolate and quantitatively characterize myeloid cells in the studies using mouse or rat models. However, the application of flow cytometry depends on commercial fluorescence-labeled antibodies against specific proteins. The biggest problem that restricts the application of flow cytometry in the studies using zebrafish models is the lack of these antibodies against zebrafish. One solution to this problem is to purify specific cells by other methods that do not depend on antibodies. For example, a DNA-staining fluorescent dye, DRAQ5, the primitive erythrocytes could be labeled and then isolated through flow cytometry. Similarly, neutrophils could be distinguished by the combinate of lectin *Phaseolus vulgaris* erythroagglutinin (PHA-E) and DRAQ5 (Konno et al. 2020). Recently, another method of antibody-free flow cytometry isolation of blood cells in zebrafish has been reported, which combines fluorescent fish lines and specific DNA-staining dyes (Konno et al. 2022). This technology is expected to be widely applied. It largely facilitates the application of flow cytometry to the research using zebrafish models and provides another option for the detection of specific gene expression levels, especially when failing to label their mRNAs through routinely used in situ hybridization assays due to the cumbersome steps and possible signal degradation (Wu et al. 2021).

With photopharmacological approach, the first conformationally strained visible light photoswitches (CS-VIPs) have been recently developed as inhibitors of the histone methyltransferase MLL1 (KMT2A) in zebrafish. Through the implantation of CS-VIP 8, in vivo bistable control of hematopoiesis could be fulfilled under visible-light irradiation with unprecedented stability (Albert et al. 2022). Regrettably, in this elegant work, Albert et al. evaluated only erythropoiesis through *o*-dianisidine staining. Promisingly, at least in theory, this technology can also be applied to the optical control of myelopoiesis, if only myeloid cells could be detected through some conventional techniques like WISH, Sudan black staining, and Neutral red staining. In addition, a novel algorithm CellComm emerges and is applied to investigate how the AGM microenvironment dictates HSPC emergence in zebrafish through cell-cell crosstalk (Lummertz da Rocha et al. 2022). As reviewed above, myelopoiesis involves the interaction between HSPC and their niche as well as complex regulatory networks. Hence, CellComm may be applied more specifically in myelopoiesis, if only performed on the data collected from specific position (i.e.*,* the CHT region) in a specific myelopoietic stages (i.e., definitive myelopoiesis). It seems that with the development and application of the technologies in bioengineering, bioinformatics, multi-omics, and other disciplines, myelopoiesis research might be even more dependent on zebrafish models in the future.

Because of the relevance and complexity of life activities, genetic manipulations often cause cascade reactions. Therefore, the results of studies using zebrafish models can hardly be directly applied to clinical health care. But it’s not just bad news. It is recently reported that a recurrent gain-of-function *ARAF* mutation was found in a 12-year-old boy with advanced anomalous lymphatic disease unresponsive to conventional therapy. Researchers replicated this mutation in zebrafish and successfully validated the functional relevance of this mutation and lymphatic phenotype. An MEK inhibitor was then screened out to rescue the anomalous phenotype in this mutant zebrafish line. Given this known, MEK inhibitor administration improved the patient’s symptoms dramatically (Li et al. 2019). In this case, the advantages of the zebrafish model were fully exploited. Zebrafish embryos that had been precisely genetically edited clearly showed how the development of lymphatic vessels was disrupted in real time under the microscope and proved the effectiveness of an MEK inhibitor in vivo. Most excitingly, these experimental results obtained on zebrafish models had achieved magical effects after being applied to the treatment of the patient. In this case, the life-saving zebrafish genetic model provides an invaluable “bench to bedside” experience, and the encore in myelopoietic diseases is full of expectation.

## Conclusions

Because of its unique advantages, zebrafish has become a well-recognized model organism for myelopoiesis studies. For decades, the application of zebrafish models has provided new insight into the cognition of myelopoietic physiology and pathophysiology, the correlation between genotype and myelopoietic phenotype, and the exploration of treatment options for myelopoietic diseases. Through precise modeling of specific genetic mutation-caused myelopoietic diseases and high-throughput screening of clinically approved drugs in zebrafish, the customization of treatment schemes for individual patient with myelopoietic disease could be practical, rapid and economical. We envisage that in the next few years zebrafish, as a convenient tool for myelopoiesis research, will more emerge in the translational medicine research on therapeutic drugs for human myelopoietic diseases and actually save more patients’ lives.

## Data Availability

Not applicable.

## Abbreviations

AGM: Aorta-gonad-mesonephros
ALM: Anterior lateral mesoderm
AML: Acute myeloid leukemia
C/EBP: CCAAT/enhancer-binding proteins
CHT: Caudal hematopoietic tissue
CML: Chronic myeloid leukemia
CMP: Common myeloid progenitors
Csf1r: Colony-stimulating factor 1 receptor
CS-VIP: Conformationally strained visible light photoswitches
DA: Dorsal aorta
DNMT1: DNA methyltransferase 1
EC: Endothelial cells
EHT: Endothelial hematopoietic transition
EMP: Erythroid-myeloid progenitors
GCSF: Granulocyte colony-stimulating factor
HEC: Hemogenic endothelial cells
hpf: Hours post fertilization
HSC: Hematopoietic stem cells
HSPC: Hematopoietic stem/progenitor cells
IDH: Isocitrate dehydrogenase
JMML: Juvenile myelomonocytic leukemia
MCSF: Macrophage-stimulating factor
MD: Myelodysplastic syndrome
MPC: Myeloid progenitor cells
MPN: Myeloproliferative neoplasm
MSC: Mesenchymal stem cells
PBI: Posterior blood island
Ph: Philadelphia Chromosome
RNF4: Ring finger protein 4
SOCS: Suppressors of cytokine signaling
SSC: Spermatogonial stem cells
STUbL: SUMO-targeted ubiquitin E3 ligase
SUMO: Small ubiquitin-related modifier
TR: Telomerase RNA component
VDA: Ventral wall of the dorsal aorta
